# Psychosocial support for HIV serodiscordant couples

**DOI:** 10.1080/21642850.2022.2084098

**Published:** 2022-06-16

**Authors:** Constance Matshidiso Lelaka, Idah Moyo, Livhuwani Tshivhase, Azwihangwisi Helen Mavhandu-Mudzusi

**Affiliations:** a Institute for Gender Studies, University of South Africa, Pretoria, South Africa; b Department of Health Studies, University of South Africa, Pretoria, South Africa; c Department of Nursing Sciences, Sefako Makgatho Health Sciences University, Pretoria, South Africa; d Department of Health Studies: Office of Graduate Studies and Research, University of South, Pretoria, South Africa

**Keywords:** HIV, HIV serodiscordant couple, psychosocial support

## Abstract

**Introduction:**

South Africa has the largest HIV epidemic, with 8.2 million people living with the virus. It has a high HIV prevalence of 13.7% and 230,000 new infections in 2020. It is estimated that HIV serodiscordant couples contribute up to 60% of new HIV infections in sub-Saharan Africa. However, there have been no specific programmes/activities to deliberately cater for couples in HIV serodiscordant relationships. The purpose of this study was to examine the psychosocial support provided for HIV serodiscordant couples both in health care settings and in the community.

**Methods:**

An interpretative phenomenological analysis (IPA) design was utilised for this study. In-depth interviews were conducted with thirteen HIV serodiscordant couples. Data collection was guided by an interview guide. All audio-recorded interview data were transcribed verbatim into written text. Data analysis was conducted using an interpretative phenomenological analysis framework. A third person-an expert in qualitative research, acted as an independent co-coder and conducted the open coding of each transcript.

**Findings:**

The findings indicated that HIV serodiscordant couples received psychosocial support from their partners, family, and health care workers. This support was emotional, or in the form of counselling, reminders on taking medication, financial and household chores. The support provided by health care providers proved to be deficient and did not address the diverse needs of this group.

**Conclusion:**

Psychosocial support plays a critical role in enhancing the quality of life of HIV serodiscordant couples. Therefore, client centred, and tailor-made interventions should be made available to this special with diverse needs group.

## Introduction

The term ‘HIV serodiscordant relationship’ refers to a sexual relationship in which one partner is HIV positive and the other partner is HIV negative (World Health Organisation [WHO], 2012). The Global AIDS Update of the Joint United Nations Programme on HIV/AIDS (UNAIDS) (2019) stipulates that in 2018 there were 1.7 million new HIV infections worldwide, adding up to a total of 37.9 million people living with HIV (PLWH). The UNAIDS (2021) reported that in 2020 sub-Saharan Africa was home to two thirds (67%) of people living with HIV globally.

According to Statistics South Africa (2021), South Africa has the largest HIV epidemic in the world. In South Africa, an estimated 8.2 million people (all ages) were living with HIV and the country had an HIV prevalence rate of 13.7% in 2021 (Statistics South Africa, 2021). In addition, UNAIDS (2021) estimated the number of new HIV infections in South Africa to 230,000 in 2021. A large proportion of new HIV infections in Africa occurs in stable heterosexual relationships, though most HIV prevention programmes in Africa focus on encouraging people to reduce the number of casual sexual partners they have, encouraging the use of condoms during casual sex and increasing fidelity among married partners (Muwonge et al., 2018). HIV serodiscordant couples are at an increased risk of HIV transmission (Davies, Matthews, Crankshaw, Cooper, & Schwartz, 2017).

The UNAIDS (2021) reported that in 2020, 65% of all new infections were from key populations and their partners. In addition, Chihana et al. (2021) indicate that HIV-serodiscordant couples contribute 30% of all new infections in sub-Saharan Africa. Related to this, Ayele, Tegegne, Damtie, Chanie, and Mekonen (2021) state that heterosexual transmission within serodiscordant relationships is one of the major sources of new HIV infections, however, there is low condom use among these couples. The UNAIDS highlight that HIV/AIDS knowledge among women may be especially low (UNAIDS, 2020). Seidu et al. (2021) recommended advocating for women education, improving socioeconomic status and empowering them to negotiate for safer sex in the fight against HIV and other sexually transmitted infections

Psychosocial refers to the dynamic relationship between the psychological and social dimension of a person and these two influence each other. The psychological aspects of development refer to an individual’s thoughts, emotions, behaviours, and perceptions, whilst the social aspects address interaction and relationships among the individual, family, peers, and community. Psychosocial refers to the close connection between psychological aspects of human experience and the wider social experience The psychosocial support then refers to the processes and actions that promote the holistic wellbeing of people in their social world. In addition, the psychosocial support activities are meant to address the psychological and social stressors associated with living with HIV (McNatt, Boothby, Wessells, & Lo, 2018).

Focused on education and counselling, psychosocial support can be associated with the following positive outcomes: increased rates of disclosure, greater treatment adherence, reduction in unprotected sex as well as HIV transmission (Mashaphu, Burns, Wyatt, & Vawda, 2018). In addition, utilisation of psychosocial interventions such as counselling, cognitive behavioural therapy, and peer support have proved to be effective in improving the mental health and overall well-being of people living with HIV (Okonji, Mukumbang, Orth, Vickerman-Delport, & Van Wyk, 2020). Such psychosocial support interventions are specifically meant to promote HIV disclosure and communication, address feelings of emotional-related distress, and challenges associated with HIV serodiscordancy (Schotanus-Dijkstra et al., 2017). Evidence has demonstrated that HIV-infected partners in sero-discordant relationships may experience high levels of stress because of unique hurdles emanating from this type of relationship. With this background, the provision of psychosocial support becomes critical (Mwakalapuka, Mwampagatwa, Bali, Mwashambwa, & Kibusi, 2017; Reed et al., 2021). Study findings by Okonji et al. (2020), demonstrated that communities play a vital role in providing psychosocial support for people living with HIV. Mukumbang et al. (2019) reported that family/household centred services were found to enhance family cohesion and communication, and such enhances shared responsibility of caring for people living with HIV. The family centred services improve communication, stimulate social support, and promote mental health among the PLWHIV. This implies that if family and community support is strengthened, then the health of PLWHIV could be improved.

Individuals testing HIV-positive or obtaining HIV-discordant results for the first-time experience unique psychological challenges. According to Kumwenda et al. (2019) in a study in Malawi, post-test psychosocial support plays a significant role in managing relationship dynamics and may be beneficial in enhancing both individual and collective coping ability and resilience towards HIV serodiscordant results in a couple. The lack of support structures has a negative impact on the health of HIV serodiscordant couples since they are exposed to high-risk behaviour as partners. Similar results were obtained by Matovu et al. (2021), who found that HIV-negative partners in discordant relationships are at risk of HIV infection. Pedrosa et al. (2016) postulate that for couples in HIV serodiscordant relationships, the main sources of social support were from friends and family. Evidence has demonstrated that in couples where another partner is living with HIV and the other is HIV negative, partner support plays a critical role (Ware et al., 2015). The authors found that where couples were in stable relationships, partners were a major source of adherence support.

Inadequate support of HIV serodiscordant couples may contribute to the failure to achieve a reduction of new HIV infections among HIV serodiscordant couples, which will defeat the target of zero new HIV infections by 2030 (UNAIDS, 2020). The aim and objectives of this study were to examine the forms of psychosocial support provided for HIV serodiscordant couples in HIV prevention, care and treatment facilities and in the community. This paper gives an insight on different types of psychosocial support provided to clients in HIV serodiscordant relationships in South Africa.

## Methods

### Study design

An interpretative phenomenological analysis design was used to explore and describe the HIV-discordant couples’ experiences pertaining to living in HIV serodiscordant relationships, the type of support provided at a regional hospital in the Gauteng Province and to develop recommendations to enhance service provision and support for this group. The focus of the design is to uncover and understand what a lived experience means to the individual through a process of in depth reflective inquiry. The IPA, therefore, an interpretative process between the researcher and researched (Noon, 2018). In the context of IPA, researchers consider the participant as the experiential expert, they acknowledge that experience can be effectively explored through a process of rich in-depth engagement and interpretation involving both the researcher and researched (Noon, 2018; Smith & Osborn, 2015).

This study utilised a phenomenological approach to explore and describe an individual’s experiences regarding the phenomenon of interest specific phenomenon (Ally, 2017). The researcher chose descriptive phenomenological because of its main reliance on in-depth interviews with individuals who have experienced the phenomenon of interest (Polit & Beck, 2021). This approach was followed because it allowed participants to narrate their experiences and in return allowed the researcher to construct the meaning of participant’s experiences relating to living in HIV serodiscordant relationships, and the type of support provided.

### Study setting

The setting for this research was one of the urban, regional specialist hospitals in Region B, Gauteng, South Africa. It is a public hospital that provides comprehensive healthcare services such as treatment of non-communicable diseases, HIV and TB-related treatment, and the provision of care and other support services. The hospital houses a few non-governmental organisations that provide services relating to HIV/AIDS, TB/MDR-TB, medical male circumcision and clinical trials to a variety of patients. The hospital is an accredited antiretroviral (ARV) treatment initiation and ongoing treatment site. The HIV clinic provides HIV counselling and testing services to individuals and couples.

### Population and sampling

The population of this study were HIV serodiscodant couples that attend Region B Hospital for their HIV services. The study sample consisted of 13 participants who were in an HIV serodiscordant relationship, that were recruited using a purposive sampling following referral by the counsellor at the facility The researcher used own judgement in selecting those participants that met the inclusion criteria as follows: heterosexual HIV-serodiscordant couples who have been in stable relationships for at least 6 months, aged 18 and above, living in Soweto and willing to participate in the study (Polit & Beck, 2021). The study excluded participants who met the inclusion criteria but could not speak either local languages (Zulu, Sotho or Xhosa) or English because the researcher would not have conducted interviews effectively. The sample size was determined by data saturation. Data saturation is reached at the stage where no new themes emerge, only redundancy of data already collected (LoBiondo-Wood & Haber, 2021; Saunders et al., 2018).

### Data collection

Data were collected using in-depth and open-ended, face-to-face interviews that were audio recorded and transcribed verbatim. Data collection was guided by an interview guide, that had open-ended questions with possible probes for further clarification. All study participants were interviewed by the researcher [first author], who is experienced in qualitative research. The purpose of in-depth, phenomenological interviews was to explore and describe experiences of HIV serodiscordant couples regarding the phenomenon under study. To ensure privacy and confidentiality, the clients were always interviewed in a selected private room where participants were accessed. To facilitate authenticity and a clear audit trail, the interviews were audio recorded and later transcribed. The researchers also kept field notes. Individual interviews were conducted by the first author between 1 December 2017 and 28 January 2018. The duration of each interview was about 30–45 min. The researchers greeted each participant, explained the purpose of the study as well as interview and obtained a written informed consent before proceeding with the data collection and audio-recording of the sessions. Research participants were encouraged to keep all information shared during the interview session confidentially. The researcher stopped collecting data when no new information emerged from the study participants, confirming data saturation (Polit & Beck, 2021).

### Data analysis

All interviews were recorded, translated, and transcribed verbatim using Microsoft Word within 48 h of data collection. Data were analysed using the interpretative phenomenological analysis (IPA) framework (Smith & Osborn, 2015).

Interpretative phenomenological analysis (IPA) was followed in order to gain a better understanding of participants experiences of living in serodiscordant relationships. Noon (2018) asserts that IPA is good in exploring lived experiences by drawing from concepts of phenomenology, hermeneutics and idiography. Analysis and interpretation of data were done following the steps as outlined by Smith and Osborn (2015) and were:(1) reading and re-reading the transcript; (2) note taking and developing emergent themes; (3) clustering the emergent themes; (4) crafting a master table of themes composed of superordinate themes, subthemes and extracts from the interviews; (5) examining and comparing the similarities between the master tables of the themes and (6) compiling a single master list composed of a superordinate theme, themes and subthemes. Each of the researchers read the transcripts and listened to the audio-recordings several times. Analysis following IPA was done by two researchers separately. The fourth author who is an experienced qualitative researcher acted as an independent coder who coded each of the independent transcripts. All the researchers met and compared the list of superordinate themes, themes, and sub-themes. A consensus was then reached after discussions to complete a table of themes as outlined in Table 2.

### Ethical measures

Protecting the rights of the participants is considered a significant ethical issue (Grove, Burns, & Gray, 2017). Therefore, all relevant ethical aspects for the study were adhered to as follows. Ethical approval was obtained from the Ethics Committee of the Department of Health at the University of South Africa (Reference number: HSHDC/6072017) prior to data collection. Permission to conduct the study was also obtained from the Department of Health-Gauteng Province before the commencement of the study. Participants were also informed that participation was voluntary and that the interviews were going to be audio recorded. In addition, participants were also informed that they were free to decline or discontinue participation in the study at any time, if they wish so, without penalty. Confidentiality and anonymity were assured by the use of pseudonyms and storage of data in a password-protected computer. The study participants were provided with a detailed information sheet that explained the details of the study and they gave a written informed consent after assimilation of essential information.

## Findings

Thirteen participants who were in an HIV serodiscordant relationship, aged between 30 and 62 years participated in the study. Eight participants were interviewed as couples which made them four couples. The remainder (five) were females, HIV positive were interviewed as individuals. Of the nine HIV positive participants, the majority (seven) were women. These participants had been in a relationship for a period of two to ten years. The researcher observed that female participants who were interviewed with their male partners were not open and could not freely share their experiences. On the other hand, females that were interviewed as individuals were open, vocal, and able to express their experiences. Table 1 outlines the demographic characteristics of the participants.

**Table 1.** Demographic data of study participants.

Pseudonym	Age in years	Interviewed as a couple/ individual	Relationship status	Number of children	Gender	HIV status	Employment status	Educational level
Noma	62	Individual	Married	3	Female	Positive	Unemployed	Grade 11
Busi	35	Individual	Single	2	Female	Positive	Employed	Tertiary
Mzie	35	Couple	Single	2	Male	Negative	Employed	Tertiary
Zamo	35	Couple	Single	2	Female	Positive	Employed	Tertiary
Linda	37	Couple	Married	2	Female	Negative	Unemployed	Grade 11
Themba	49	Couple	Married	2	Male	Positive	Employed	Grade 11
Thembi	39	Couple	Single	0	Female	Negative	Employed	Tertiary
Vusi	57	Couple	Single	5	Male	Positive	Self-employed	Grade 12
Lwazi	30	Individual	Single	1	Female	Positive	Self-employed	Tertiary
Rosy	30	Couple	Single	1	Female	Positive	Unemployed	Grade 12
Thato	35	Couple	Single	0	Male	Negative	Self-employed	Grade 12
Bongi	45	Individual	Married	2	Female	Positive	Employed	Tertiary
Thando	42	Individual	Single	1	Female	Positive	Self-employed	Grade 12

Data analysis reported two superordinate themes, together with several categories or sub-themes on the support provided to participants in HIV serodiscordant relationships both at institutional and community level settings. Table 2 illustrates the results regarding the support provided to these participants.

**Table 2.** Summary of superordinate-theme, themes, and sub-themes.

Superordinate themes	Themes	Sub-themes
Institutional level support	Support from service providers	HIV treatment services (ART Medication)
		HIV Counselling services
		Educational information
Community level support	Support from the family providers	Emotional support
		Financial support
		Home/house chores
		Reminder about medication
		Disclosure support
	Support by partner	Emotional support
		Accompany to the clinic for consultation/collect medication
		Reminder to take medication
		Assistance for house chores

## Superordinate theme 1: institutional level support

This theme focusses on the support provided to the HIV serodiscordant couples by the counsellors. The support received by HIV serodiscordant couples will be discussed under the following sub-themes: HIV treatment (antiretroviral medication), HIV counselling, and information received.

### Theme 1.1: support from service providers

Counsellors play a significant role in the lives of patients presenting with HIV related health challenges. They are the first point of contact in most healthcare facilities, offer various services to patients, and encourage them to freely access the services without any fear of being judged. Patients are seen and referred accordingly where possible. Participants understood the importance of improving the quality of their health and were able to consult to access healthcare services. The services received, amongst others include HIV treatment, HIV counselling, and information.

#### Sub-theme 1.1.1: HIV treatment services (ART medication)

Participants also indicated that counsellors displayed professionalism and that in addition to receiving treatment, they felt they were accepted and treated with respect. This is supported by the following quotes:

I’m at attending XX clinic, and they helped me so much. They accepted me, gave me treatment/medication and they are very professional. (Noma)

They give me medication all the time when I need it. I am also happy with the treatment that I receive from the HIV clinic. They treat me well, they accepted me, they support me, and they give me medication all the time when I need it. (Themba)

Participants also acknowledged receiving a comprehensive package of HIV care and treatment services as reflected by the extract below:

I normally get additional tests such as viral load test, pap-smear, high-blood, and blood sugar since I am diabetic. (Rosy)

#### Sub-theme 1.1.2: HIV counselling services

Participants regarded their service providers as ‘loving and respectful’ and highlighted the extent of general satisfaction they had from the services provided. The provision of counselling was noted as one of the key services provided by counsellor’s providers. The following extracts illustrate that:

They provided me with support; the counselling was great, and I felt happy at the same time. I had the opportunity to express myself and I was able to ask questions concerning my health. I was provided with advice on how I should look after myself. (Zamo)

I like their service; they treat people well and with respect. I received counselling session and this assited me to further provide support to my partner. (Mzie)

They helped me a lot to understand HIV and gave me more information. Then I went to attend the adherence clinic, that’s where they taught us about the medication and CD4 for how is low and how is high and the meaning. (Thando)

While some participants were happy with the counselling services, others felt the counselling services were either inadequate or inappropriate as shown by the extract below:

I counselled myself and accepted, they need to provide more counselling and support to such people like us. When I found out about my HIV status, I was blank, and I was not comfortable, I did not get any counselling or support on my status. If there can be information and education for couples about discordancy, that would help us to improve our level of understanding, and the support will make us to cope better because of knowing what is happening. (Busi)

Some participants felt that besides getting medication, healthcare providers do not provide any specific attention to HIV serodiscordant couples as reflected by the quote below:

I never receive much; I only go and get my medication. There was a time when it was not taking medication, and my viral load was high. The healthcare provider was mad at me, and that’s when I only received counseling. Other than that, there is nothing except medication and that adherence counselling. (Lwazi)

#### Sub-theme 1.1.3: educational information

Participants also commented on how counsellors use queues to disseminate vital information and communicate with HIV serodiscordant couples as shown by the following extracts:

They do health talks a lot while we are waiting in the queues, they talk about illness and inform and educate us, they encourage us to do additional tests like prostate cancer, sugar diabetes, high blood pressure, etc. (Vusi)

Another client had this to say:

They educated me, and they shared information with me and taught me how to take medication correctly. (Themba)

## Superordinate theme 2: community level support

### Theme 2.1: support from family members

It was evident that a support system in the lives of HIV serodiscordant couples was important. Most HIV serodiscordant couples received various types of support systems from various family members, and they were grateful to receive it.

Couples received positive support from their family members. This category produced sub-categories which relate to the types of support services received by HIV serodiscordant couples from their families. For partners who have disclosed to some or all their family members, support came in many ways. In times of distress, for instance, serious sickness, couples admitted that their families provide them with emotional support.

#### Sub-theme 2.1.1: emotional support

According to the interviewed couples, close family members were the pillars of their emotional support, especially in times of distress. Such times include the period of just finding out about their own or their partner’s HIV positive status. While in the time of denial, disbelief, and loss of confidence in the relationship, family members provided the support as illustrated by the extracts below:

My mom understands and accepted me and my relationship, she helps me a lot with my younger child, and she is my pillar of strength. (Bongi)

My sister and my mother love and support me so much. They check on me constantly, regarding my pre-exposure prophylaxis medication, health and how I am coping. They ask a lot of questions about me and my health and not about my partner. Even me too, I tell them how I feel, and they reach out to me when I need their support or help. They also love my family, that is my partner, and my children. I feel welcome when I visit my family, and I am not scared. I feel at home. (Thato)

In general terms, my parents and sister do provide me with support and we are all in good terms. They are normally there, and they have been there for me. They have ensured my health is taken care of. (Thembi)

In addition to family social support received, some participants reported that they had the support of friends as well, which added a positive impact in their lives. This is supported by the following quotes:

My friend also knows about my HIV status, and she supports me so much and encourages me to stay positive. (Vusi)

#### Sub-theme 2.1.2: financial support

In addition to emotional support, participants also reported having received some instrumental support from their family members such as financial support.

Mom sometimes gives me money for taxi fare when accompanying my partner to the clinic for consultation/treatment. (Linda)

#### Sub-theme 2.1.3: home/house chores support

The family was also noted as playing a key role in taking care of home chores where one partner was not able to. Such chores include daily household chores as well as taking care of children, as shown in the quotes below:

She is responsible for a lot of things in the family, like taking care of the children, loving and supporting us all the time, fix things that are not going well, doing the washing and when I go to work, I am clean. (Themba)

Caregiving was cited as a key support service offered by family members as they provided not only care for children but also came to offer support in times of more serious sickness as attested to below:

My mom understands the situation and my relationship, she helps me a lot with my younger child, and she is my pillar of strength. She takes care of my child, and I don’t pay her. The other thing is that mom helps me with house chores particularly when I am not feeling well. I am happy with her continued support. (Busi)

#### Sub-theme 2.1.4: reminder about medication

Other family members offered their support by giving infected individuals constant reminders for taking medication as well as staying healthy. The following two quotes show that:

With the family, everyone supports me, and they remind me of taking medication, even now my mom knows I'm coming to Limpopo and she just told me that I must not forget my medication. (Zamo)

She reminds me to take my medication most of the time; I am so happy for the support that I get from her. (Vusi)

#### Sub-theme 2.1.5: disclosure support

Because of limited understanding of HIV-serodiscordancy, participants that had supportive families opted to do selective disclosure as illustrated by extracts below. The responses quoted below show selective disclosure:

I am not comfortable about it; I think it better if it’s only my parent and sister who know about my partner’s HIV positive status because they are supportive. I am scared; I am also scared of the stigma. We kept this a secret for many years. (Thembi)

### Theme 2.2: support from partner

One of the most crucial pillars leading to the happiness of couples living in HIV-serodiscordancy is support for each other. Most respondents reported a positive support system for each other and how they were coping.

#### Sub-theme 2.2.1: emotional support

The interviewed couples generally admitted their partners offered the greatest support in times of need and all the time. Emotional support came in the form of partners being present for each other, not taking the HIV infection too seriously and staying together with their partner despite their HIV status. The two quotes below attest to that:

I accepted and supported her. I am happy with that. After testing HIV positive, I provided her with a lot of support, and she appreciated the support. (Thato)

My partner was very supportive and did not stress too much about it. So, we handled this together as a team and were there for each other. (Bongi)

However, a small number of interviewed individuals reported that their partners provided limited support to them and seemed less interested in getting involved; hence they wanted their partners to become more supportive as attested to by the quote below:

My wishes are; if he (partner) can be open, talk lots more about it (HIV serodiscordant), and ask questions about how I am feeling, he’s not open about those things. If I am down, he must ask, and he must be open when talking to me because he knows my status and sees me taking the medication. He doesn’t ask about my medication like now I am taking treatment for TB prevention medication; he doesn’t ask why I am taking more than three pills instead of 1 pill. He doesn’t ask anything. (Thando)

In a gesture of total support, some uninfected partners tend to engage in unprotected sex with their partners to show that they did not mind getting infected at all, and were fine with their partners’ condition as shown by the quote below:

I do not care if I get HIV or what, I want to be with her and marry her. Even if I can get infected, I will still be with her, and I will join her and start taking medication I do not care. Should I get HIV, HIV infected or not, I am with her, I love her. (Mzie)

#### Sub-theme 2.2.2: accompanying to clinic for consultation/collect medication

Partners were found to accompany their infected partners to hospitals, attended full counselling sessions, and any discussions that come.

She comes to the clinic with me and goes with me in all stations from point 1 to point 2, all the station. (Thato)

On top of supervising medicine consumption, partners sometimes keep track of their infected partners’ hospital visit schedule to ensure they never miss appointments as attested to by the following quote:

Even now, he is sometimes supervising to ensure that I take my medication well and also to ensure I attend my visits. (Rosy)

However, in other instances, HIV negative partners in the relationship stay in denial and finding it hard to be open to talk about HIV with their partners. Such partners end up not talking about medication or even accompanying their respective partners to hospital appointments. One interviewee had this to say:

He does not remind me to take any medication or remind me of any clinic appointments, that one is my responsibility. (Lwazi)

#### Sub-theme 2.2.3: reminder to take medication

As the infected partners may forget taking their medication or doctors’ appointments, couples noted that they or their partners constantly came in to offer support.

I am not always at home; she will call me to tell me to take my medication closer to the time. She reminds me that it’s closer to 9pm as it is medication time. (Themba)

In the majority of times, he finds time to remind me of taking my medication. (Thando)

To ensure that their partners take medication, other couples noted that they become involved in the medication taking process to ensure that their infected partners take all required medication.

My partner was supervising me so much at the time, even now he is sometimes supervising to ensure that I take my medication well and to ensure that I attend my visits. He sometimes sees when I am taking my medication. (Noma)

As busy as we are at home, he finds time to remind me of taking my medication. He calls me aside and talks to me about medication. (Bongi)

#### Sub-theme 2.2.4: assistance with house chores

Sharing responsibilities was key among partners. They voluntarily and willingly made sure they assist with household chores as they arise without waiting for the other party, thus showing valued support to their partners.

He has been very helpful to the kids and me. He has been very hands-on with the house chores although he’s not a good cook he tries his best. When it comes to packing things in the house, he is very good as he ensures the house is neat. He is very helpful, and he assists me so much. He also cleans the house. He is good. I can rely on him in so many things, and he is not disappointing. He delivers. (Rosy)

My partner is so supportive. Sometimes it’s quite hectic at home when we have events, but he is there to support me. I do not feel any stress as he is doing a lot of things so because of him, I do not usually get stressed at all. (Busi)

## Discussion

This study found that counsellors, who were the first point of contact at the study setting played crucial role in providing support for couples in HIV serodiscordant relationships. The healthcare workers provided support in the form of counselling and information giving, however, some participants felt the counselling was not person centred and not addressing issues related to HIV serodiscordancy. Related to this, Morton et al. (2021) identified key barriers to engaging with couples in HIV testing services and these included concerns about trust in the relationship, lack of open discussion during counselling sessions. The authors emphasise the need for optimising couple-focused interventions

The findings of this study further confirm the importance of a support system even outside the healthcare environment. For participants that disclosed their HIV status or HIV serodiscordancy, family members offered them different forms of support. It also emerged that in critical moments for example in times of sickness or some distress, family members provided support. This study found that partner support was one of the important pillars in HIV serodiscordant couples. Support provided by partners and family was as follows: emotional, financial, accompaniment to the clinic for collecting medication, medication reminders and assistance with house chores. The discussion will focus on key findings and their implications, varying forms of support, challenges, and recommendations.

This study brings unique insights into the psychosocial support received by HIV serodiscordant couples in the South African context. It was noted in this study’s findings that since support play a significant role in the lives of HIV serodiscordant couples, both the infected and affected individual reported having received various support services from the service providers, family members, and from each other as partners. This support followed selective disclosure on HIV status by study participants. These findings concur with those of a study by Matovu et al. (2021), in Uganda indicating that the serodiscordant couples usually seek support from health workers, friends and relatives. Related to this, Dessalegn et al. (2019) in a study in Ethiopia, found that HIV disclosure for individual in serodiscordant relationships was sometimes delayed, with the likelihood for onward transmission. In addition, the major barrier to disclosure was fear of negative outcomes such as verbal abuse and physical violence.

Services received by participants in this study from the healthcare service providers included HIV counselling, HIV treatment and educational information to empower them on how to deal with the health challenges facing HIV serodiscordant couples. Furthermore, support services reported by counsellors are also recommended by the CDC-HIV-prevention bluebook (2019); the provision of an early HIV treatment with antiretroviral medicines not only improves the health of people with HIV but also reduces the risk of transmitting the virus to the other partner. Similarly, a study by Reed et al. (2021) established that PLWH in sero-discordant sexual partnerships demonstrated improved uptake of ART.

One of the drivers of HIV transmission in Sub-Saharan Africa is HIV sero-discordancy among married or cohabitating partners and contribute a significant proportion of new infections (Irungu et al., 2016; Patel et al., 2018). A recent meta-analysis showed greatly reduced HIV transmission in serodiscordant couples in whom the HIV positive partner is virally suppressed (Oldenburg et al., 2016). Furthermore, according to CDC-HIV-prevention bluebook (2019) a combination of antiretroviral therapy has been shown to reduce the rate of new HIV infections to an uninfected partner by 96%.

Whilst counselling was considered by some participants as comprehensive, the other participants felt the counselling was general and did not centre on issues relating to serodiscordance. This is contrary to the recommendations from other studies that have demonstrated that couples-focused counselling or educational programmes were associated with reduced HIV transmission, or some behaviour modification such as reduced unprotected sex (Mashaphu et al., 2018). Related to this, research has demonstrated that provision of psychosocial support to couples during the post-test counselling session plays an integral role and improves the general wellbeing, enhances effective management of HIV infections and relationship dynamics of couples in serodiscordant relationships (Kumwenda et al., 2019; Mashaphu & Burns, 2017). Our ﬁndings concur with those of other studies that highlight the fact that specific interventions that address the diverse and unique needs of people in mixed HIV-status relationships is lacking (Mthembu, Khan, & Manengela, 2018). The authors call for the need to strengthen sero-discordant interventions as a response to managing the HIV epidemic among this special group of clients. In addition, Mashaphu and Burns (2017) emphasise the importance of such interventions as: couple-centred HIV prevention services, treatment as prevention and tailor made sexual health services to quality of life for people in serodiscordant relationships.

Counselling and the provision of in-depth educational information are crucial at any point along the treatment process to assist couples to get through their most troubling times and arm them with the necessary information. This is consistent with study findings by Doherty et al. (2016) who asserts that the purpose of the pre-test counselling is to give someone who is considering being tested for all the necessary information and support to make an informed decision. Findings from a study by Wall et al. (2019) demonstrated that couple counselling in HIV care settings is a high impact strategy to prevent transmission and ultimately improve clinical outcomes. This is further corroborated by Wall et al. (2017), who argues that counselling plays a critical role in dealing with psychological needs of the individuals in HIV serodiscordant relationships.

Family members are a crucial link in the treatment process of an HIV positive individual. Their support helps to create peace of mind and a sense of belonging, which will divert an HIV positive person’s attention from daily troubles to personal wellbeing. Among such support systems are emotional support, which was highly valued by couples who participated in this study. During their period of severe distress after obtaining results, family members were often there to offer support. This is similar to findings from other studies; for instance, Okonji et al. (2020) who believe that social support is an important factor for health maintenance. Kumala et al. (2022) established that family social support has a positive influence on people living with HIV as they face stress related to health-disease process, contributing towards the treatment against HIV and dealing with aspects related to the fear of death. Pedrosa et al. (2016) assert that the relationship with the health professional and the support received by the patients impacts positively on treatment compliance. Another form of support was material support in the form of money for rentals, food, and other necessities.

With individuals dealing with HIV, house chore support offered by family members has proven important. In most cases, siblings or mothers of the HIV positive individuals would be readily available for laundry work, cooking, cleaning and other activities as corroborated by Xu et al. (2017) who noted that family member support includes assisting with taking care of the house chores, preparing meals and spending time with the patient. Chaudhury et al. (2016) in a study in Rwanda further noted that family-based support interventions had a positive influence on people living with HIV or affected with HIV as they face stress related health-disease process, contributing towards the treatment against HIV and dealing with aspects related to the fear of death. This is further emphasised in studies by Reis et al. (2019) and Yombi and Mertes (2018) that indicate that counselling and guidance about sexual HIV prevention for people HIV serodiscordant relationships couples are critical components in HIV care. Okonji et al. (2020) also stressed that psychosocial support is an important variable in the prevention of diseases, promotion of health, therapeutic compliance, and in the process of recovery from illness.

A study by Morton et al. (2021) identified key barriers to engaging with couples in HIV testing services and these included concerns about trust in the relationship, lack of open discussion during counselling sessions. The authors emphasise the need for optimising couple-focused interventions

As chores arise in the house, it becomes up to the HIV negative partner to make sure they are attended to and completed on time. Regardless of being a husband or wife, the HIV negative partner find themselves as the main breadwinner for the house, especially at times where the HIV positive partner is incapacitated and cannot work. Goldenberg and Stephenson (2015) also identified assistance with chores by partners as important. These findings relating to partner support in a variety of ways suggest that at each stage of continuum care, a provider’s focus beyond a biomedical approach that encourages partner-specific psychological support may assist with improving adherence to treatment.

The Department of Health, South Africa (2016) and WHO (2020), emphasise the effectiveness of pre-exposure prophylaxis (PrEP) in HIV serodiscordant couples. While in support of the use of PrEP, Muessig and Cohen (2014) noted that up to 90% of HIV positive patients who took daily pills are less likely to transmit the disease to their partners during intercourse, and therefore couples are more likely to bear children successfully without both horizontal and vertical transmission. In the case of this study, the opposite was found since there was no specific counselling that focused on HIV prevention for the negative partner and no mention about HIV prevention services such as PrEP and voluntary medical male circumcision.

The findings of this study showed that some participants disclosed their HIV status to a select few confidants, one or two family members due to fear of stigma and discrimination. These findings concur with those of a study conducted in South Africa by Madiba, Ralebona, and Lowane (2021), who posit that disclosure to family and friends about one’s HIV status is usually deliberate, selective, and often planned, where PLWHIV tend to weigh the risks and benefits associated this planned disclosure. Related to this, study findings by Antonini et al. (2021) emphasise the importance of professional counselling and psycho-social support in assisting couples to cope with HIV-discordance and related disclosure.

Disclosing HIV status was advocated for by a participant of the study only to her mother and sister in sought of support but not to relatives in fear of being stigmatised. Since the participant was fearing the stigma attached to disclosing the status, she resorted to selective disclosure to those she believed could support her. Ambissa, Sendo, Assefa, and Guta (2021), advocated for disclosure as a means that led to safer sex practices, prevention of re-infection, social and emotional, financial and encourages partners to make informed reproductive health choices. On the other hand, Poku, Owusu, Mullen, Markham, and McCurdy (2017) argue that disclosure could bring challenges such as conflict with partner as well as abuse and withdrawal of finances.

## Conclusion and recommendations

The findings of this study underscore the need to design specific interventions that address the diverse and challenging psychosocial needs of HIV serodiscordant couples. From the study, it is clear that the quality of life and treatment outcomes are dependent on the support they receive from both the health care settings and their social networks. The need to support and empower these couples on issues related to discordant relationships remains critical. The study has established that the unique needs of serodiscordant couples are largely not addressed in HIV prevention, care and treatment programmes. To enhance the quality of care and treatment outcomes, it is critical that the psychosocial landscape and related dynamics of couples in HIV serodiscordant relationships be understood. It is further recommended that future studies should include both partners in serodiscordant relationships to better understand differential risk-management strategies and relationship building aspects. In view of the fact that the counselling provided at the study setting was found to be deficient, the researchers strongly recommend the development of a psychosocial model that would be utilised in providing counselling for couples in serodiscordant relationships. The authors also recommend strengthening of the quality of counselling through provision of differentiated person centred counselling services. This could be further enhanced through clinical competency training for healthcare workers that addresses communication and individualised counselling.

In order for the country to contribute to the control of the HIV epidemic, that is aimed at ending AIDS by 2030, it is critical that effective and comprehensive HIV prevention, care, and treatment services are provided to HIV serodiscordant couples. These services should be tailor made for them, for example access to PrEP for the HIV negative partner in a serodiscordant relationship. In addition to this, the researchers recommend that health care providers be trained in knowledge and skill to necessary support the specific needs of this. Since HIV serodiscordant couples had a support system from each other, it is important that continuous education pertaining to serodiscordacy be shared with the couples in healthcare facilities so that they can continue to improve their knowledge and manage the challenges they face.

## Limitation of the study

This study was conducted in one regional hospital. Since a qualitative research method was employed for this study, the results of the study cannot be generalised to other provinces with HIV serodiscordant couples.
